# Multivariate visualization of the global COVID-19 pandemic: A comparison of 161 countries

**DOI:** 10.1371/journal.pone.0252273

**Published:** 2021-05-28

**Authors:** Jane K. L. Teh, David A. Bradley, Jack Bee Chook, Kee Huong Lai, Woo Teck Ang, Kok Lay Teo, Suat-Cheng Peh

**Affiliations:** 1 School of Mathematical Sciences, Sunway University, Selangor, Malaysia; 2 School of Engineering and Technology, Sunway University, Selangor, Malaysia; 3 School of Medical and Life Sciences, Sunway University, Selangor, Malaysia; 4 PEMANDU Associates, Kuala Lumpur, Malaysia; National University of Sciences and Technology (NUST), Islamabad, PAKISTAN

## Abstract

**Background:**

The aim of the study was to visualize the global spread of the COVID-19 pandemic over the first 90 days, through the principal component analysis approach of dimensionality reduction.

**Methods:**

This study used data from the Global COVID-19 Index provided by PEMANDU Associates. The sample, representing 161 countries, comprised the number of confirmed cases, deaths, stringency indices, population density and GNI per capita (USD). Correlation matrices were computed to reveal the association between the variables at three time points: day-30, day-60 and day-90. Three separate principal component analyses were computed for similar time points, and several standardized plots were produced.

**Results:**

Confirmed cases and deaths due to COVID-19 showed positive but weak correlation with stringency and GNI per capita. Through principal component analysis, the first two principal components captured close to 70% of the variance of the data. The first component can be viewed as the severity of the COVID-19 surge in countries, whereas the second component largely corresponded to population density, followed by GNI per capita of countries. Multivariate visualization of the two dominating principal components provided a standardized comparison of the situation in the161 countries, performed on day-30, day-60 and day-90 since the first confirmed cases in countries worldwide.

**Conclusion:**

Visualization of the global spread of COVID-19 showed the unequal severity of the pandemic across continents and over time. Distinct patterns in clusters of countries, which separated many European countries from those in Africa, suggested a contrast in terms of stringency measures and wealth of a country. The African continent appeared to fare better in terms of the COVID-19 pandemic and the burden of mortality in the first 90 days. A noticeable worsening trend was observed in several countries in the same relative time frame of the disease’s first 90 days, especially in the United States of America.

## 1. Introduction

A local outbreak of viral pneumonia of unknown cause in Wuhan, China was initially reported to the WHO on 31 Dec 2019 [1]. Within a few weeks, the coronavirus (COVID-19) was discovered in other continents and worldwide. This led to the WHO declaring the novel coronavirus outbreak a public health emergency of international concern (PHEIC). Although deaths due to the coronavirus were confined to China at that point of time, confirmed cases escalated rapidly, especially in the European continent. By the middle of March 2020, Europe had more reported cases and deaths than the rest of the world combined, except for China. With rising levels of spread and severity, as well as inaction in many countries, WHO declared COVID-19 a pandemic on March 11 2020 [1].

The WHO has urgently called on all countries to take immediate action to contain the alarming spread of the virus and minimize the impact on all sectors [1]. Particularly, stringent nationwide measures were recommended to prevent the spread of the COVID-19 disease and flatten the curve of new cases. Governments all over the world have heeded the call and taken measures which include closure policies, restrictions in movement, and testing regimes. However, different levels of stringency were implemented in different countries. For instance, Singapore and South Korea have been more proactive and aggressive in tackling the pandemic than others [2,3].

When examining the correlation between stringent measures and the incidence of COVID-19 cases, the population density and wealth of a country are potential confounding factors [4]. However, studies of the association between the spread of COVID-19 and population density have not shown consistent findings. The spread of COVID-19 may not be directly related to the density of a country [5], but in certain parts of the world such as in India and Algeria, there exists a moderate to strong association between the two [6,7]. On the other hand, the wealth of a country does play an important role in reducing the spread of COVID-19, as it determines the bargaining power in attaining resources, such as personal protective equipment for health-care workers and ventilators for patients [8]. Yet, there is evidence suggesting that the situation in poorer countries is better, as COVID-19 mortality rate is still highly concentrated in high-income countries [9].

In this context, there has been a strong interest in examining the rapid spread of the COVID-19 pandemic [10,11], which has resulted in huge impact on human lives and the economy [12]. This study aims to employ a multivariate approach to visualize the global spread of the COVID-19 pandemic, also seeking to understand how the disaster has spread since the first confirmed cases in countries worldwide. We attempt to describe the global situation of the proliferation of COVID-19 across time, by considering the cases and deaths caused by COVID-19, the stringency measures taken by countries, and the possible confounding factors that may work against these measures.

## 2. Methods

In this section, we describe the dataset of the study, and the principal component analysis technique as a suitable tool to achieve the objectives of this study.

### 2.1 Dataset

We acquired the complete data from the Global COVID-19 Index (GCI) provided by PEMANDU Associates [13]. The dataset consists of confirmed incidences and deaths due to COVID-19 cases, and stringency indicators for the first 90 days, starting with the first confirmed cases reported for 161 countries (and special administrative regions). This dataset also includes the data for population density and GNI per capita (USD). The list of countries can be found in S1 Table.

The GCI engine extracted the daily number of COVID-19 cases and deaths from the COVID-19 Data Repository provided by the Center for Systems Science and Engineering (CSSE) at Johns Hopkins University. Population density and GNI per capita (USD) for each country were obtained from the World Bank and were updated as of July 2020. Stringency indicators for the first 90 days were obtained from the University of Oxford. The Oxford COVID-19 Government Response Tracker (OxCGRT) systematically collects information on the stringency of government responses to the pandemic on 18 indicators such as school closures and travel restrictions [14]. Data from these indicators are aggregated into a set of four common indices reporting a number between 1 and 100, which indicates the strictness of government policies.

### 2.2 Data analysis

Principal component analysis (PCA) is a robust multivariate technique for dimensionality reduction [15,16]. Supposing that there are *n* observations with measurements on a set of *p* variables, the PCA technique derives the principal components by finding a sequence of linear combinations of the variables, *X*_1_,*X*_2_,…,*X_p_*, that have maximal variance, and are mutually uncorrelated [16]. The first principal component, *Z*_1_, is the direction in space along which projections have the largest variance. The second principal component, *Z*_2_, is the direction which maximizes variance among all directions, and is orthogonal to the first, *Z*_1_. Algebraically, the first two principal components are given by the following formulae:

$$Z_{1}=\phi_{11}X_{1}+\phi_{21}X_{2}+\cdots +\phi_{p1}X_{p}$$

$$Z_{2}=\phi_{12}X_{1}+\phi_{22}X_{2}+\cdots +\phi_{p2}X_{p}$$

This technique is based on the decomposition of the original data matrix into the scores and loadings matrices [16]. The score values (e.g. for *Z*_1_: *z*_11_,…,*z*_*n*1_) classify the samples, whereas the loading values (e.g. for *ϕ*_1_: *ϕ*_11_,…,*ϕ*_*p*1_) classify the variables in terms of their separation of the samples. Each of the loadings is referred to as the weight for each variable when calculating the principal components, and the loading vector is unique up to a sign flip [16].

PCA uses the dependencies between variables to produce a low-dimensional representation of a dataset, while preserving as much information as possible [15,16]. It also serves as a tool for data visualization, as it retains trends and patterns, and transforms the data into fewer dimensions. Trends and correlations which are “hidden” in the data can be visualized and described in principal component space. More details about the principal component analysis technique can be found elsewhere [15,16].

For each of the 161 countries, these variables were used in the following analyses: 1) number of confirmed COVID-19 cases on day-30, day-60 and day-90; 2) number of deaths due to COVID-19 cases on day-30, day-60 and day-90; 3) stringency indicator on day-30, day-60 and day-90; 4) population density; and 5) GNI per capita (USD). Due to skewness in the data, the data was shifted and log-transformed.

We computed three correlation matrices on three time points: day-30, day-60 and day-90. This was followed by three separate principal component analyses on similar time points. The PCA technique was performed using the “prcomp” command of the R statistical software, after standardizing each variable to have mean zero and standard deviation of one. Standardization involves rescaling the variables such that each will have the properties of a standard normal distribution with a mean of zero and a standard deviation of one. Several plots were produced from the PCA analysis using the “ggplot2” and “ggrepel” R packages [17].

## 3. Results

### 3.1 Correlation matrices

Correlation values are quite similar on all three different time points (Table 1). Confirmed cases and deaths due to COVID-19 show strong positive correlation (~ 0.90) with each other. Both confirmed cases and deaths variables are positively correlated with stringency and GNI per capita, but the strength of the associations are rather weak (0.20–0.40).

**Table 1:** Correlation matrix.

**Day-30**					
	**Pop.Den**	**GNI**	**Con.30**	**Dth.30**	**Str.30**
Pop.Den (*Population density*)	1.000	0.168	0.038	0.021	-0.132
GNI (*GNI per capita*)	0.168	1.000	0.329	0.214	-0.198
Con.30 (*Confirmed cases day 30*)	0.038	0.329	1.000	0.881	0.239
Dth.30 (*Deaths day 30*)	0.021	0.214	0.881	1.000	0.256
Str.30 (*Stringency day 30*)	-0.132	-0.198	0.239	0.256	1.000
**Day-60 **					
	**Pop.Den**	**GNI**	**Con.60**	**Dth.60**	**Str.60**
Pop.Den (*Population density*)	1.000	0.168	0.032	0.012	-0.034
GNI (*GNI per capita*)	0.168	1.000	0.347	0.347	0.067
Con.60 (*Confirmed cases day 60*)	0.032	0.347	1.000	0.914	0.258
Dth.60 (*Deaths day 60*)	0.012	0.347	0.914	1.000	0.247
Str.60 (*Stringency day 60*)	-0.034	0.067	0.258	0.247	1.000
**Day-90 **					
** **	**Pop.Den**	**GNI**	**Con.90**	**Dth.90**	**Str.90**
Pop.Den (*Population density*)	1.000	0.168	0.029	-0.002	-0.056
GNI (*GNI per capita*)	0.168	1.000	0.279	0.316	0.011
Con.90 (*Confirmed cases day 90*)	0.029	0.279	1.000	0.920	0.335
Dth.90 (*Deaths day 90*)	-0.002	0.316	0.920	1.000	0.272
Str.90 (*Stringency day 90*)	-0.056	0.011	0.335	0.272	1.000

### 3.2 Proportion of variance explained by principal components

On all three time points, PCA analysis produced somewhat similar proportions of variance explainable by the five principal components (Table 2). The variance of the data contributed by the first component is between 42–45%, and the variance contributed by the second component is between 22–27%. Taken together, the first two components can explain around 68% of the variance in the data. Of the remaining variance, the third component captures around 17%, the fourth around 14%, and the fifth around 2%.

**Table 2:** Proportion of variance explained from PCA analysis.

Proportion of Variance	PC1	PC2	PC3	PC4	PC5
PCA Day-30	0.42	0.27	0.17	0.12	0.02
PCA Day-60	0.45	0.22	0.17	0.14	0.02
PCA Day-90	0.44	0.23	0.17	0.14	0.02

### 3.3 Principal component loading vectors

There appear to be similar patterns in the first loadings on all three time points (Table 3). The first loading vector places large, positive, and approximately equal weights on confirmed cases and deaths. The weights for stringency and GNI per capita are also positive but relatively smaller. Hence, this first component seems to correspond more towards an indication of the level of severity of the COVID-19 surge in the country. Countries with large positive scores on the first component would indicate very serious COVID-19 situations at country level.

**Table 3:** Principal component loading vectors from PCA analysis.

**Day-30**					
	**PC1**	**PC2**	**PC3**	**PC4**	**PC5**
Pop.Den	0.051	0.530	0.846	-0.032	-0.003
GNI	0.291	0.580	-0.356	0.663	0.107
Con.30	0.662	-0.004	-0.047	-0.195	-0.722
Dth.30	0.647	-0.074	-0.003	-0.331	0.683
Str.30	0.237	-0.614	0.394	0.641	0.021
**Day-60**					
	**PC1**	**PC2**	**PC3**	**PC4**	**PC5**
Pop.Den	0.066	0.796	0.461	0.387	-0.016
GNI	0.375	0.441	-0.142	-0.803	0.003
Con.60	0.626	-0.082	-0.155	0.277	0.708
Dth.60	0.624	-0.094	-0.178	0.269	-0.706
Str.60	0.274	-0.396	0.843	-0.239	-0.010
**Day-90**					
	**PC1**	**PC2**	**PC3**	**PC4**	**PC5**
Pop.Den	0.043	0.720	0.637	0.269	-0.033
GNI	0.317	0.541	-0.334	-0.703	0.033
Con.90	0.634	-0.065	-0.057	0.296	0.709
Dth.90	0.630	-0.042	-0.155	0.292	-0.702
Str.90	0.315	-0.428	0.674	-0.510	-0.053

As for the second loading vector, population density and GNI per capita have positive projections whereas stringency has negative projections. The weight for stringency, on day-30 appears to be the largest among these three variables, indicating that countries with large negative scores on the second component had more stringent measures implemented. Results on day-60 and day-90 however, show that the weight for population density is the largest. Hence, the second component at later time points appear to be largely driven by the population density of the country. Countries with large positive scores on the second component would most notably indicate higher population density, followed by GNI per capita.

The third loading vector on day-30 also has a different loading pattern as compared to later time points. Results on day-30 indicate that the third component is largely driven by population density, whereas on day-60 and day-90, the component corresponds more towards stringency measures. On the contrary, the fourth and fifth loading vectors are similar on all three time points. The fourth component appears to be driven more by the GNI per capita of the country. Lastly, the fifth loading vector places large and approximately equal weights on confirmed cases and deaths, but both variables have opposing directions.

### 3.4 Principal component score vectors

**Fig 1:**
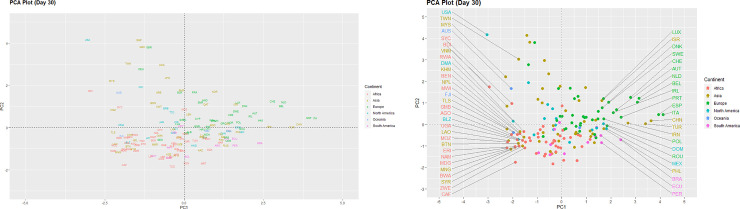
PCA plot (Day 30).

**Fig 2:**
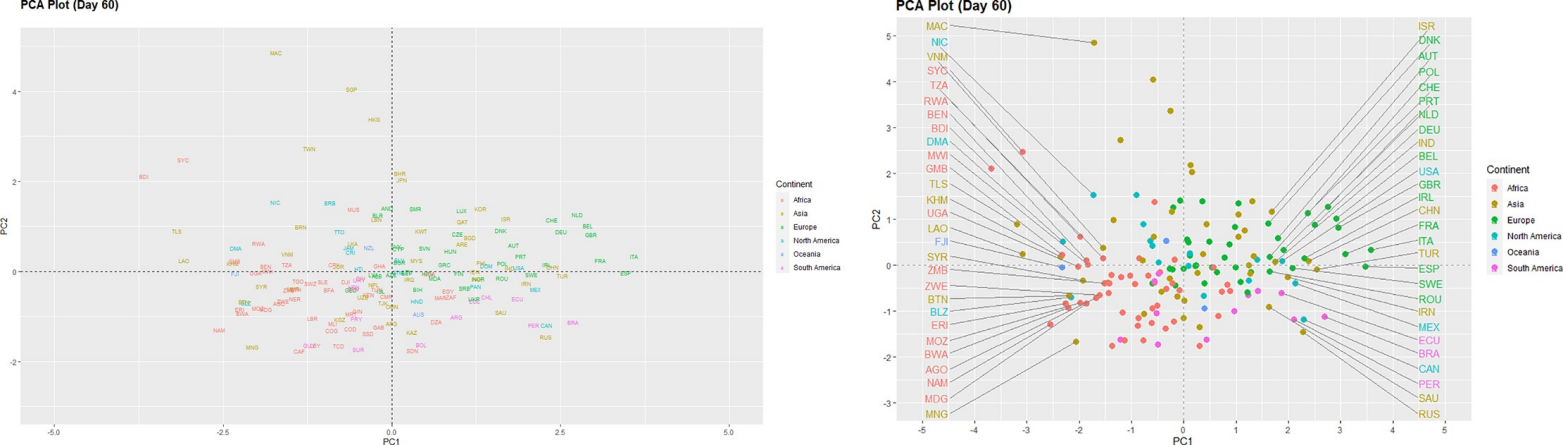
PCA plot (Day 60).

**Fig 3:**
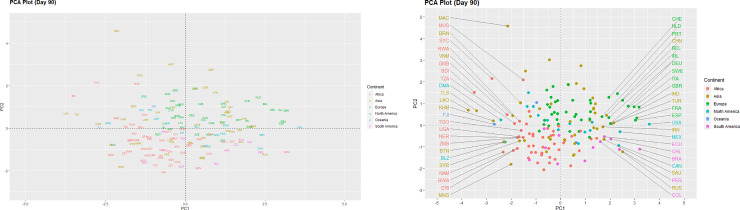
PCA plot (Day 90).

We can examine differences between the countries on the three different time points via the two principal component score vectors as shown in Figs 1–3. These low-dimensional representations preserve an adequate amount of information, as the first two components capture close to 70% of the variance of the data [16]. The International Organization for Standardization (ISO) codes for countries are displayed as coloured text and represent the scores for the first two principal components. Country coordinates in the plots can be found in S2 Table.

Overall, most countries appear close to the origin (0,0), indicating somewhat moderate levels of severity in the COVID-19 pandemic (Fig 1A and 1B). Countries such as Italy, Spain, China and Turkey have large positive scores on the first component and appear at the far right of the plot. This indicates that these countries were experiencing a highly severe COVID-19 surge at that point of time. On the other hand, countries with large negative scores such as the United States of America and Burundi were in a less severe COVID-19 situation. Most countries from the African continent appear to cluster below the origin (0,0) of the plot, indicating perhaps more stringent government response to the pandemic, lower GNI per capita as well as population density.

Large positive scores for Italy and Spain on the first principal component indicate that the situation had not improved for both countries on day-60 (Fig 2A and 2B). Moreover, there appears to be a growing cluster of countries from the European continent at the far right of the plot, which includes France, United Kingdom, Belgium, Germany, Switzerland and the Netherlands. It is also notable that more countries from the other continents have now appear towards the right side of the origin (0,0), suggesting a surge in severity since day-30 of the COVID-19 pandemic. These countries include the United States of America, Australia and Malaysia. Conversely, Macau (China) and Singapore appear at the top left quadrant of the plot, indicating higher population density and a less severe COVID-19 situation.

Similar cluster of countries can be seen on day-90 of the pandemic (Fig 3A and 3B). Most countries from the African continent appear towards the left side of the origin (0,0), whereas the majority from the European continent are towards the right side. Though, the United States of America has emerged as the new epicentre of the COVID-19 pandemic.

## 4. Discussion

The availability of a consolidated, comprehensive dataset mapped on a day-by-day basis has allowed us to track the world-wide spread of COVID-19 from the first human case to have been determined (with for the moment general accord lent to this) right through to day-90 of its evolution. In so doing, the intention has been to seek emergent patterns, “hidden” within several influencing factors. Our study attempts to visualize the surge of the COVID-19 pandemic through the principal component analysis approach of dimensionality reduction, and provides snapshots on three different time points of the pandemic. Our findings reveal relationships between countries, continents, and the influencing factors.

Compared with previous studies, our findings indicate the unequal severity of the spread of COVID-19 in different countries [10,11]. In addition, our visualization of the global spread of COVID-19 shows the unequal severity of the pandemic across continents and over time. Findings reveal rather distinct patterns in clusters of countries, separating many European countries from those in Africa. Overall, the African continent appears to fare better in terms of the COVID-19 pandemic and the burden of mortality over the first 90 days, possibly due to the relatively less equipped health, track and trace infrastructure [18]. However, the opposite can be seen among countries from Europe, particularly for a select few, such as Italy, Spain and France. These findings appear to corroborate with the timeline of WHO’s response to COVID-19 [1]. Although the first COVID-19 outbreak occurred in China, Italy has quickly overtaken China in infections and deaths [19]. As the COVID-19 pandemic surges, its heavy toll remained concentrated in Europe [9].

Consistent with ideas of others, our multivariate analysis indicate an inverse association between stringent nationwide measures, and the country’s population density and GNI per capita [4]. Our findings further suggest a delineation of countries in terms of these factors. A contrast can be seen, especially between many countries from the African and European continents, indicating that developed and wealthier countries are more severely affected by the pandemic as compared to their poorer and less developed counterparts [9]. This observed contrast is likely attributed to higher population density and international human mobility, which has been facilitated by globalization among European countries [7,20–22]. Taken collectively, these reasons may have propelled the spread of the COVID-19 pandemic in the continent.

On the contrary, the COVID-19 pandemic has been less severe in Africa [9,23]. A plausible reason for the relatively low mortality burden in Africa is that most African governments have adopted quick and strict measures to mitigate the spread of COVID-19. Moreover, there has been substantial public support despite these strict public health and social measures [23]. Other potential reasons for the less severe COVID-19 surge in Africa are likely due to the generally younger age demographics in Africa [9], and the relatively warmer climate [21,23].

Overall, the other continents do not appear to have fared too badly. One contributing reason may be that several of these countries have adequate capacities and mechanisms for emergency response, as guided by the Asia Pacific Strategy for Emerging Diseases and Public Health Emergencies (APSED) [24]. As evidence has shown among countries such as Singapore and South Korea [3,25], rapid detection together with vigorous contact tracing are effective in mitigating the spread of COVID-19 disease. Though, a slight contrast can be seen in other countries from the similar region such as Indonesia and India [26]. The cause may be due to widespread poverty in these countries, and insufficient testing and health-care infrastructures.

Although COVID-19 was first identified in Wuhan, China, the Chinese government’s strict contact tracing and lockdown measures have effectively mitigated the spread of the pandemic [27,28]. Conversely, the outbreak in the United States has been relentless since its first confirmed COVID-19 case. As was reported, the COVID-19 pandemic had spread to all 50 states by the middle of March in 2020, and there were more than 5,000 COVID-19 associated deaths by early April 2020 [29]. Fuelled by widespread community transmission, the COVID-19 pandemic has surged exponentially in the United States.

There are limitations in this study. Not all countries have been included in the multivariate analysis. This is due to reasons such as incomplete data on stringency indicators for the first 90 days, and statistical indicators on population density and GNI per capita (USD) as provided by the World Bank are not the latest as of July 2020 for several of these countries.

It is also worth noting that the OxCGRT tracks the policy measures implemented by the various governments around the world [14]. This stringency indicator does not reveal the public compliance and enforcement capabilities, which can be made more complex based on the governance structures and also the available infrastructure. Governments may implement countermeasures to contain the spread of the COVID-19 disease, but they are not necessarily able to sufficiently track compliance to these measures. For instance, strict measures have not effectively mitigated the spread of the disease in countries such as India and Bangladesh [30]. Moreover, the effectiveness of these measures also depends on public and private sector compliance and cooperation to reduce community transmission [24,30]. All in all, stricter governance and immense political will are required to rapidly implement aggressive countermeasures to mitigate widespread community transmission [31].

For future research, there is merit in looking into further granularity, not least within large countries, including those which have independent governance mechanisms in different states. That apart, incorporating variables denoting proportions of the older generation may also reveal interesting findings. Thus far, numerous studies have been done to model the transmission of COVID-19 in various parts of the world [32–34], as the pandemic continues to wreak havoc on human lives. Future research may build upon our findings through the extraction of principal components to be applied in machine learning models, for making predictions of the spread of COVID-19.

## 5. Conclusion

This study utilises principal component analysis, an unsupervised learning technique, in order to provide a visual representation of relationships between countries and several influencing factors. The objective of this study is not to provide conclusive results. Instead, given a multivariate combination of factors, we have produced standardized visual comparisons of 161 countries, and improved on the interpretability of the global spread of the COVID-19 pandemic over the first 90 days.

The COVID-19 outbreak has been a pandemic of historic proportions. At the time of the writing of this paper, the pandemic continues to be a threat to humanity in its entirety. The devastating impact on lives, livelihoods and social disruption felt all over the world will forever etch the COVID-19 pandemic into the annals of history. The global community should draw upon lessons learnt from this COVID-19 pandemic, and be alert and prepared for the next infectious disease outbreak—lest history repeats itself.

## Supporting information

S1 Table List of countries. (DOCX)

S2 Table Country coordinates in PCA plots. (DOCX)
