# Supplementary material for: Multivariate visualization of the global COVID-19 pandemic: A comparison of 161 countries
Source: PLoS One. 2021 May 28;16(5):e0252273. doi: 10.1371/journal.pone.0252273 (PMC8162616; doi:10.1371/journal.pone.0252273)
Supplement: S1 Table — (DOCX) [file pone.0252273.s001.docx]

**S1 Table. List of Countries**

| **No.** | **Country ISO Code** | **Country** | **Continent** |
| --- | --- | --- | --- |
| 1 | AFG | Afghanistan | Asia |
| 2 | AGO | Angola | Africa |
| 3 | ALB | Albania | Europe |
| 4 | AND | Andorra | Europe |
| 5 | ARE | United Arab Emirates | Asia |
| 6 | ARG | Argentina | South America |
| 7 | AUS | Australia | Oceania |
| 8 | AUT | Austria | Europe |
| 9 | AZE | Azerbaijan | Europe |
| 10 | BDI | Burundi | Africa |
| 11 | BEL | Belgium | Europe |
| 12 | BEN | Benin | Africa |
| 13 | BFA | Burkina Faso | Africa |
| 14 | BGD | Bangladesh | Asia |
| 15 | BGR | Bulgaria | Europe |
| 16 | BHR | Bahrain | Asia |
| 17 | BIH | Bosnia and Herzegovina | Europe |
| 18 | BLR | Belarus | Europe |
| 19 | BLZ | Belize | North America |
| 20 | BOL | Bolivia | South America |
| 21 | BRA | Brazil | South America |
| 22 | BRB | Barbados | North America |
| 23 | BRN | Brunei | Asia |
| 24 | BTN | Bhutan | Asia |
| 25 | BWA | Botswana | Africa |
| 26 | CAF | Central African Republic | Africa |
| 27 | CAN | Canada | North America |
| 28 | CHE | Switzerland | Europe |
| 29 | CHL | Chile | South America |
| 30 | CHN | China | Asia |
| 31 | CIV | Cote d'Ivoire | Africa |
| 32 | CMR | Cameroon | Africa |
| 33 | COD | Congo (Kinshasa) | Africa |
| 34 | COG | Congo (Brazzaville) | Africa |
| 35 | COL | Colombia | South America |
| 36 | CPV | Cabo Verde | Africa |
| 37 | CRI | Costa Rica | North America |
| 38 | CYP | Cyprus | Europe |
| 39 | CZE | Czechia | Europe |
| 40 | DEU | Germany | Europe |
| 41 | DJI | Djibouti | Africa |
| 42 | DMA | Dominica | North America |
| 43 | DNK | Denmark | Europe |
| 44 | DOM | Dominican Republic | North America |
| 45 | DZA | Algeria | Africa |
| 46 | ECU | Ecuador | South America |
| 47 | EGY | Egypt | Africa |
| 48 | ERI | Eritrea | Africa |
| 49 | ESP | Spain | Europe |
| 50 | EST | Estonia | Europe |
| 51 | ETH | Ethiopia | Africa |
| 52 | FIN | Finland | Europe |
| 53 | FJI | Fiji | Oceania |
| 54 | FRA | France | Europe |
| 55 | GAB | Gabon | Africa |
| 56 | GBR | United Kingdom | Europe |
| 57 | GEO | Georgia | Europe |
| 58 | GHA | Ghana | Africa |
| 59 | GIN | Guinea | Africa |
| 60 | GMB | Gambia | Africa |
| 61 | GRC | Greece | Europe |
| 62 | GTM | Guatemala | North America |
| 63 | GUY | Guyana | South America |
| 64 | HKG | China (Hong Kong) | Asia |
| 65 | HND | Honduras | North America |
| 66 | HRV | Croatia | Europe |
| 67 | HTI | Haiti | North America |
| 68 | HUN | Hungary | Europe |
| 69 | IDN | Indonesia | Asia |
| 70 | IND | India | Asia |
| 71 | IRL | Ireland | Europe |
| 72 | IRN | Iran | Asia |
| 73 | IRQ | Iraq | Asia |
| 74 | ISL | Iceland | Europe |
| 75 | ISR | Israel | Asia |
| 76 | ITA | Italy | Europe |
| 77 | JAM | Jamaica | North America |
| 78 | JOR | Jordan | Asia |
| 79 | JPN | Japan | Asia |
| 80 | KAZ | Kazakhstan | Asia |
| 81 | KEN | Kenya | Africa |
| 82 | KGZ | Kyrgyzstan | Asia |
| 83 | KHM | Cambodia | Asia |
| 84 | KOR | Korea, South | Asia |
| 85 | KWT | Kuwait | Asia |
| 86 | LAO | Laos | Asia |
| 87 | LBN | Lebanon | Asia |
| 88 | LBR | Liberia | Africa |
| 89 | LBY | Libya | Africa |
| 90 | LKA | Sri Lanka | Asia |
| 91 | LTU | Lithuania | Europe |
| 92 | LUX | Luxembourg | Europe |
| 93 | LVA | Latvia | Europe |
| 94 | MAC | China (Macau) | Asia |
| 95 | MAR | Morocco | Africa |
| 96 | MDA | Moldova | Europe |
| 97 | MDG | Madagascar | Africa |
| 98 | MEX | Mexico | North America |
| 99 | MLI | Mali | Africa |
| 100 | MMR | Burma | Asia |
| 101 | MNG | Mongolia | Asia |
| 102 | MOZ | Mozambique | Africa |
| 103 | MRT | Mauritania | Africa |
| 104 | MUS | Mauritius | Africa |
| 105 | MWI | Malawi | Africa |
| 106 | MYS | Malaysia | Asia |
| 107 | NAM | Namibia | Africa |
| 108 | NER | Niger | Africa |
| 109 | NGA | Nigeria | Africa |
| 110 | NIC | Nicaragua | North America |
| 111 | NLD | Netherlands | Europe |
| 112 | NOR | Norway | Europe |
| 113 | NPL | Nepal | Asia |
| 114 | NZL | New Zealand | Oceania |
| 115 | OMN | Oman | Asia |
| 116 | PAK | Pakistan | Asia |
| 117 | PAN | Panama | North America |
| 118 | PER | Peru | South America |
| 119 | PHL | Philippines | Asia |
| 120 | POL | Poland | Europe |
| 121 | PRT | Portugal | Europe |
| 122 | PRY | Paraguay | South America |
| 123 | QAT | Qatar | Asia |
| 124 | ROU | Romania | Europe |
| 125 | RUS | Russia | Asia |
| 126 | RWA | Rwanda | Africa |
| 127 | SAU | Saudi Arabia | Asia |
| 128 | SDN | Sudan | Africa |
| 129 | SEN | Senegal | Africa |
| 130 | SGP | Singapore | Asia |
| 131 | SLE | Sierra Leone | Africa |
| 132 | SLV | El Salvador | North America |
| 133 | SMR | San Marino | Europe |
| 134 | SRB | Serbia | Europe |
| 135 | SSD | South Sudan | Africa |
| 136 | SUR | Suriname | South America |
| 137 | SVK | Slovakia | Europe |
| 138 | SVN | Slovenia | Europe |
| 139 | SWE | Sweden | Europe |
| 140 | SWZ | Eswatini | Africa |
| 141 | SYC | Seychelles | Africa |
| 142 | SYR | Syria | Asia |
| 143 | TCD | Chad | Africa |
| 144 | TGO | Togo | Africa |
| 145 | TJK | Tajikistan | Asia |
| 146 | TLS | Timor-Leste | Asia |
| 147 | TTO | Trinidad and Tobago | North America |
| 148 | TUN | Tunisia | Africa |
| 149 | TUR | Turkey | Asia |
| 150 | TWN | Taiwan | Asia |
| 151 | TZA | Tanzania | Africa |
| 152 | UGA | Uganda | Africa |
| 153 | UKR | Ukraine | Europe |
| 154 | URY | Uruguay | South America |
| 155 | USA | United States of America | North America |
| 156 | UZB | Uzbekistan | Asia |
| 157 | VEN | Venezuela | South America |
| 158 | VNM | Vietnam | Asia |
| 159 | ZAF | South Africa | Africa |
| 160 | ZMB | Zambia | Africa |
| 161 | ZWE | Zimbabwe | Africa |
