# Supplementary material for: Multivariate visualization of the global COVID-19 pandemic: A comparison of 161 countries
Source: PLoS One. 2021 May 28;16(5):e0252273. doi: 10.1371/journal.pone.0252273 (PMC8162616; doi:10.1371/journal.pone.0252273)
Supplement: S2 Table — (DOCX) [file pone.0252273.s002.docx]

**S2 Table. Country Coordinates in PCA Plots**

| **No.** | **Country ISO Code** | **Day - 30** | | **Day - 60** | | **Day - 90** | |
| --- | --- | --- | --- | --- | --- | --- | --- |
|  |  | **PC1** | **PC2** | **PC1** | **PC2** | **PC1** | **PC2** |
| 1 | AFG | -0.24 | -1.30 | 0.00 | -1.15 | 0.52 | -1.40 |
| 2 | AGO | -1.59 | -0.75 | -1.67 | -0.71 | -1.11 | -0.96 |
| 3 | ALB | 0.17 | -0.20 | -0.21 | -0.09 | -0.48 | -0.03 |
| 4 | AND | 0.66 | 1.30 | -0.07 | 1.41 | -0.56 | 1.62 |
| 5 | ARE | -0.79 | 1.67 | 1.05 | 0.62 | 1.32 | 0.74 |
| 6 | ARG | 1.06 | -0.84 | 0.96 | -1.00 | 1.31 | -1.04 |
| 7 | AUS | -2.06 | 1.67 | 0.40 | -0.94 | 0.62 | -0.81 |
| 8 | AUT | 2.23 | 0.69 | 1.81 | 0.59 | 1.21 | 1.02 |
| 9 | AZE | 0.08 | -0.25 | -0.01 | -0.07 | 0.37 | -0.29 |
| 10 | BDI | -2.95 | 1.74 | -3.68 | 2.11 | -3.49 | 1.52 |
| 11 | BEL | 3.08 | 1.03 | 2.91 | 1.01 | 2.51 | 1.15 |
| 12 | BEN | -1.70 | -0.43 | -1.86 | 0.11 | -1.39 | 0.05 |
| 13 | BFA | -0.04 | -1.09 | -0.93 | -0.41 | -1.27 | -0.39 |
| 14 | BGD | 0.91 | 0.13 | 1.16 | 0.76 | 1.34 | 0.79 |
| 15 | BGR | 0.41 | 0.08 | 0.12 | 0.21 | -0.25 | 0.59 |
| 16 | BHR | -0.07 | 1.78 | 0.13 | 2.18 | 0.31 | 1.86 |
| 17 | BIH | 0.66 | -0.46 | 0.39 | -0.40 | -0.10 | 0.05 |
| 18 | BLR | -0.14 | 1.67 | -0.21 | 1.25 | -0.13 | 1.13 |
| 19 | BLZ | -1.77 | -0.70 | -2.16 | -0.70 | -2.25 | -0.77 |
| 20 | BOL | 0.10 | -1.36 | 0.44 | -1.63 | 0.98 | -1.66 |
| 21 | BRA | 2.40 | -0.72 | 2.69 | -1.13 | 3.20 | -1.11 |
| 22 | BRB | -0.57 | 0.93 | -0.91 | 1.53 | -1.30 | 1.50 |
| 23 | BRN | -0.97 | 1.17 | -1.35 | 0.98 | -1.62 | 1.04 |
| 24 | BTN | -1.77 | -0.88 | -2.19 | -0.67 | -2.30 | -0.78 |
| 25 | BWA | -1.74 | -1.01 | -2.21 | -0.93 | -1.76 | -1.08 |
| 26 | CAF | -1.88 | -1.76 | -1.37 | -1.76 | -0.68 | -2.07 |
| 27 | CAN | 1.23 | -0.27 | 2.29 | -1.19 | 2.35 | -0.95 |
| 28 | CHE | 2.79 | 1.25 | 2.37 | 1.14 | 1.94 | 1.28 |
| 29 | CHL | 1.41 | -0.13 | 1.42 | -0.57 | 2.03 | -0.65 |
| 30 | CHN | 3.63 | 0.16 | 2.39 | 0.10 | 1.85 | 0.45 |
| 31 | CIV | -0.07 | -0.53 | -0.46 | -0.15 | -0.30 | -0.28 |
| 32 | CMR | 0.08 | -0.65 | -0.08 | -0.55 | 0.17 | -0.72 |
| 33 | COD | -0.47 | -1.42 | -0.61 | -1.27 | -0.23 | -1.58 |
| 34 | COG | -0.71 | -1.47 | -0.89 | -1.32 | -0.77 | -1.39 |
| 35 | COL | 1.36 | -0.54 | 1.23 | -0.66 | 1.60 | -0.76 |
| 36 | CPV | -0.76 | -0.09 | -0.85 | 0.15 | -0.53 | -0.16 |
| 37 | CRI | -0.23 | 0.27 | -0.61 | 0.42 | -0.54 | 0.23 |
| 38 | CYP | 0.43 | 0.43 | 0.09 | 0.50 | -0.44 | 0.84 |
| 39 | CZE | 1.48 | 0.61 | 0.97 | 0.84 | 0.49 | 1.15 |
| 40 | DEU | -1.35 | 2.78 | 2.51 | 0.89 | 2.73 | 0.88 |
| 41 | DJI | -0.28 | -0.77 | -0.68 | -0.23 | -0.30 | -0.46 |
| 42 | DMA | -1.98 | 0.15 | -2.31 | 0.52 | -2.42 | 0.25 |
| 43 | DNK | 1.91 | 1.03 | 1.61 | 0.92 | 1.19 | 1.15 |
| 44 | DOM | 1.70 | 0.03 | 1.41 | 0.12 | 1.27 | 0.14 |
| 45 | DZA | 0.81 | -1.03 | 0.67 | -1.11 | 0.75 | -1.19 |
| 46 | ECU | 1.59 | -0.51 | 1.87 | -0.61 | 1.76 | -0.54 |
| 47 | EGY | 0.75 | -0.53 | 0.84 | -0.43 | 1.23 | -0.64 |
| 48 | ERI | -1.90 | -1.03 | -2.25 | -0.84 | -1.91 | -1.17 |
| 49 | ESP | 3.94 | 0.46 | 3.47 | -0.03 | 3.02 | 0.23 |
| 50 | EST | 0.76 | 0.05 | 0.22 | -0.02 | -0.36 | 0.50 |
| 51 | ETH | -1.28 | -0.76 | -1.42 | -0.39 | -0.26 | -0.86 |
| 52 | FIN | 0.87 | 0.37 | 0.99 | -0.05 | 0.57 | 0.39 |
| 53 | FJI | -1.97 | -0.38 | -2.32 | -0.05 | -2.69 | 0.04 |
| 54 | FRA | 0.33 | 1.68 | 3.09 | 0.25 | 3.07 | 0.33 |
| 55 | GAB | -0.64 | -0.82 | -0.19 | -1.23 | -0.07 | -1.14 |
| 56 | GBR | -1.10 | 3.82 | 2.96 | 0.82 | 3.16 | 0.83 |
| 57 | GEO | -0.76 | -0.57 | -0.60 | -0.40 | -0.86 | -0.27 |
| 58 | GHA | -0.16 | -0.45 | -0.18 | 0.13 | -0.09 | -0.04 |
| 59 | GIN | -0.24 | -1.00 | -0.51 | -0.88 | -0.48 | -1.08 |
| 60 | GMB | -2.05 | -0.47 | -2.33 | 0.23 | -2.17 | 0.48 |
| 61 | GRC | 1.24 | 0.21 | 0.78 | 0.16 | 0.21 | 0.60 |
| 62 | GTM | -0.34 | -0.24 | 0.09 | -0.01 | 0.98 | -0.31 |
| 63 | GUY | -0.94 | -1.33 | -1.21 | -1.63 | -1.26 | -1.62 |
| 64 | HKG | -0.71 | 2.97 | -0.26 | 3.38 | -0.45 | 3.01 |
| 65 | HND | 0.32 | -0.84 | 0.38 | -0.66 | 0.72 | -0.86 |
| 66 | HRV | 0.58 | -0.08 | 0.53 | -0.05 | -0.12 | 0.52 |
| 67 | HTI | -1.13 | -0.49 | -0.48 | 0.06 | -0.09 | -0.21 |
| 68 | HUN | 1.00 | 0.34 | 0.87 | 0.45 | 0.63 | 0.49 |
| 69 | IDN | 1.38 | 0.19 | 1.24 | 0.00 | 1.33 | -0.12 |
| 70 | IND | 1.16 | -0.30 | 1.75 | 0.07 | 2.07 | 0.07 |
| 71 | IRL | 2.09 | 0.54 | 2.30 | 0.15 | 1.95 | 0.35 |
| 72 | IRN | 2.69 | 0.05 | 1.99 | -0.26 | 1.71 | -0.08 |
| 73 | IRQ | 0.55 | -0.44 | 0.26 | -0.17 | 0.56 | -0.45 |
| 74 | ISL | 0.01 | 0.39 | -0.16 | -0.44 | -0.68 | 0.03 |
| 75 | ISR | 1.63 | 1.10 | 1.69 | 1.17 | 1.30 | 1.25 |
| 76 | ITA | 4.13 | 0.45 | 3.59 | 0.33 | 2.97 | 0.86 |
| 77 | JAM | -0.71 | 0.27 | -0.65 | 0.53 | -0.91 | 0.45 |
| 78 | JOR | -0.16 | -0.45 | -0.79 | 0.11 | -1.23 | 0.41 |
| 79 | JPN | -0.53 | 2.36 | 0.15 | 2.03 | 0.94 | 1.68 |
| 80 | KAZ | 0.51 | -1.07 | 0.30 | -1.35 | 0.54 | -1.37 |
| 81 | KEN | -0.32 | -0.74 | -0.33 | -0.51 | 0.12 | -0.81 |
| 82 | KGZ | -0.47 | -1.25 | -0.77 | -1.07 | -0.58 | -1.22 |
| 83 | KHM | -1.70 | -0.33 | -2.35 | 0.18 | -2.73 | 0.20 |
| 84 | KOR | 0.97 | 1.71 | 1.31 | 1.39 | 0.63 | 1.92 |
| 85 | KWT | -0.79 | 1.00 | 0.43 | 0.90 | 1.39 | 0.56 |
| 86 | LAO | -2.17 | -0.76 | -3.08 | 0.24 | -3.74 | 0.70 |
| 87 | LBN | 0.15 | 0.63 | -0.23 | 1.16 | -0.39 | 0.99 |
| 88 | LBR | -0.66 | -1.38 | -1.17 | -1.04 | -1.02 | -1.26 |
| 89 | LBY | -1.04 | -1.35 | -1.13 | -1.64 | -0.45 | -1.74 |
| 90 | LKA | -0.35 | -0.06 | -0.58 | 0.62 | -0.86 | 0.75 |
| 91 | LTU | 0.76 | -0.05 | 0.23 | -0.01 | -0.15 | 0.26 |
| 92 | LUX | 1.70 | 1.20 | 1.04 | 1.35 | 0.31 | 1.88 |
| 93 | LVA | -0.20 | 0.18 | -0.27 | -0.08 | -0.66 | 0.23 |
| 94 | MAC | -1.31 | 3.82 | -1.71 | 4.86 | -2.15 | 4.58 |
| 95 | MAR | 1.15 | -0.70 | 0.72 | -0.58 | 0.44 | -0.51 |
| 96 | MDA | 0.73 | -0.31 | 0.64 | -0.15 | 0.74 | -0.30 |
| 97 | MDG | -1.91 | -1.07 | -1.86 | -0.84 | -1.18 | -1.26 |
| 98 | MEX | 1.82 | -0.20 | 2.13 | -0.40 | 2.40 | -0.30 |
| 99 | MLI | -0.40 | -1.36 | -0.87 | -1.15 | -0.76 | -1.29 |
| 100 | MMR | -0.91 | -0.78 | -1.45 | -0.38 | -1.46 | -0.71 |
| 101 | MNG | -2.08 | -1.15 | -2.06 | -1.68 | -2.01 | -1.81 |
| 102 | MOZ | -2.20 | -0.82 | -1.99 | -0.82 | -1.31 | -1.52 |
| 103 | MRT | 0.62 | -1.69 | -0.60 | -0.95 | -0.72 | -1.02 |
| 104 | MUS | 0.01 | 0.87 | -0.56 | 1.38 | -1.52 | 2.10 |
| 105 | MWI | -1.81 | -0.46 | -1.83 | 0.01 | -1.12 | -0.50 |
| 106 | MYS | -2.28 | 2.22 | 0.37 | 0.25 | 0.38 | 0.17 |
| 107 | NAM | -2.24 | -1.09 | -2.55 | -1.30 | -2.33 | -1.25 |
| 108 | NER | -0.24 | -1.27 | -1.44 | -0.61 | -1.74 | -0.57 |
| 109 | NGA | 0.03 | -0.28 | 0.56 | -0.05 | 0.81 | -0.27 |
| 110 | NIC | -1.35 | 1.94 | -1.73 | 1.54 | -1.36 | 0.86 |
| 111 | NLD | 3.14 | 1.21 | 2.75 | 1.27 | 2.29 | 1.46 |
| 112 | NOR | 1.47 | 0.25 | 1.29 | -0.15 | 0.83 | 0.24 |
| 113 | NPL | -1.54 | -0.59 | -0.27 | -0.29 | 0.16 | -0.67 |
| 114 | NZL | 0.72 | -0.15 | -0.34 | 0.53 | -0.98 | 1.04 |
| 115 | OMN | -0.76 | -0.37 | 0.01 | -0.77 | 0.56 | -0.85 |
| 116 | PAK | 1.18 | -0.56 | 1.27 | -0.18 | 1.47 | -0.16 |
| 117 | PAN | 1.41 | -0.16 | 1.24 | -0.33 | 1.19 | -0.25 |
| 118 | PER | 1.61 | -0.84 | 2.11 | -1.19 | 2.37 | -1.13 |
| 119 | PHL | 1.71 | -0.20 | 1.33 | 0.19 | 1.17 | 0.23 |
| 120 | POL | 1.71 | 0.26 | 1.64 | 0.17 | 1.20 | 0.62 |
| 121 | PRT | 2.41 | 0.34 | 1.91 | 0.33 | 1.66 | 0.39 |
| 122 | PRY | -0.46 | -0.94 | -0.52 | -1.05 | -0.62 | -1.04 |
| 123 | QAT | 0.69 | 1.11 | 1.05 | 1.11 | 1.41 | 0.99 |
| 124 | ROU | 1.84 | -0.06 | 1.64 | -0.14 | 1.18 | 0.29 |
| 125 | RUS | 1.32 | -0.85 | 2.28 | -1.45 | 2.50 | -1.24 |
| 126 | RWA | -1.55 | -0.34 | -1.98 | 0.62 | -1.66 | 0.19 |
| 127 | SAU | 1.25 | -0.44 | 1.62 | -0.91 | 1.75 | -0.65 |
| 128 | SDN | 0.07 | -1.68 | 0.31 | -1.76 | 0.41 | -1.88 |
| 129 | SEN | -0.96 | -0.60 | -0.56 | -0.38 | -0.39 | -0.49 |
| 130 | SGP | -1.42 | 4.15 | -0.59 | 4.05 | 0.82 | 2.75 |
| 131 | SLE | -0.37 | -0.87 | -1.02 | -0.22 | -1.13 | -0.41 |
| 132 | SLV | -0.26 | -0.03 | 0.12 | 0.26 | 0.37 | 0.07 |
| 133 | SMR | 0.52 | 1.20 | 0.35 | 1.39 | -0.31 | 1.79 |
| 134 | SRB | 1.34 | -0.47 | 1.08 | -0.37 | -0.22 | 1.07 |
| 135 | SSD | -0.14 | -1.06 | -0.35 | -1.38 | -0.44 | -1.48 |
| 136 | SUR | -0.49 | -1.42 | -0.50 | -1.73 | -0.12 | -1.80 |
| 137 | SVK | -0.07 | 0.38 | 0.07 | 0.56 | -0.53 | 0.95 |
| 138 | SVN | 0.92 | 0.32 | 0.49 | 0.51 | -0.39 | 1.29 |
| 139 | SWE | 1.64 | 0.78 | 2.08 | -0.07 | 2.01 | 0.07 |
| 140 | SWZ | -0.98 | -0.43 | -1.20 | -0.27 | -0.72 | -0.57 |
| 141 | SYC | -2.03 | 0.97 | -3.09 | 2.48 | -2.78 | 2.15 |
| 142 | SYR | -1.60 | -1.01 | -1.93 | -0.33 | -1.66 | -0.74 |
| 143 | TCD | -0.37 | -1.83 | -0.78 | -1.64 | -0.94 | -1.74 |
| 144 | TGO | -1.32 | -0.62 | -1.38 | -0.21 | -1.60 | -0.25 |
| 145 | TJK | 0.58 | -0.36 | -0.13 | -0.69 | -0.41 | -0.65 |
| 146 | TLS | -2.23 | -0.22 | -3.19 | 0.89 | -3.40 | 0.66 |
| 147 | TTO | -0.38 | 0.73 | -0.77 | 0.89 | -1.11 | 0.86 |
| 148 | TUN | 0.35 | -0.61 | -0.22 | -0.40 | -1.27 | 0.63 |
| 149 | TUR | 3.40 | 0.02 | 2.54 | -0.09 | 2.14 | 0.10 |
| 150 | TWN | -1.76 | 3.04 | -1.21 | 2.73 | -1.43 | 2.50 |
| 151 | TZA | -0.87 | -0.24 | -1.55 | 0.16 | -1.86 | 0.07 |
| 152 | UGA | -1.81 | -0.71 | -2.02 | -0.03 | -1.70 | -0.53 |
| 153 | UKR | 1.39 | -0.62 | 1.22 | -0.60 | 1.17 | -0.57 |
| 154 | URY | -0.09 | 0.09 | -0.48 | -0.19 | -0.67 | -0.17 |
| 155 | USA | -3.05 | 4.17 | 1.88 | 0.09 | 3.58 | 0.04 |
| 156 | UZB | -0.13 | -0.77 | -0.42 | -0.57 | -0.48 | -0.56 |
| 157 | VEN | -0.29 | -0.23 | -0.56 | -0.35 | -0.07 | -0.47 |
| 158 | VNM | -2.24 | 0.84 | -1.54 | 0.38 | -1.95 | 0.43 |
| 159 | ZAF | 0.66 | -0.48 | 0.88 | -0.56 | 1.46 | -0.61 |
| 160 | ZMB | -1.47 | -0.81 | -1.52 | -0.40 | -1.51 | -0.56 |
| 161 | ZWE | -1.51 | -1.09 | -1.61 | -0.65 | -1.29 | -0.97 |
